# Functional analysis of differently expressed ferroptosis-related genes in patients with mitral valve prolapse

**DOI:** 10.3389/fgene.2022.1062212

**Published:** 2022-11-29

**Authors:** Hui Xie, Liushun Wang, Yihu Tang, Meng Zhao, Zihao Wang, Mingzhu Liu, Quangong Zhao, Jingxin Zhou, Yanhu Wu

**Affiliations:** Department of Cardiovascular Surgery, Nanjing Medical University First Affiliated Hospital, Nanjing, China

**Keywords:** valve disease, mitral value prolapse, ferroptosis, lncRNA, gene

## Abstract

**Background:** The prevalence of mitral valve prolapse (MVP) in heart valvular diseases is globally increasing. However, the understanding of its etiology and pathogenesis is limited. So far, the relationship between ferroptosis-related genes and long non-coding RNAs (lncRNAs) in MVP remains unexplored. This study investigates the potential pathogenesis of ferroptosis-related genes in MVP and provides a therapeutic target for the disease.

**Methods:** Blood samples from patients with MVP and healthy volunteers were collected for transcriptomic sequencing to analyze the expression of ferroptosis-related differentially expressed genes (DEGs) and differentially expressed long non-coding RNAs (DElncRNAs Co-expression network of ferroptosis-related DEGs and DElncRNAs. Furthermore, this work conducted GO and KEGG enrichment analyses.

**Results:** *CDKN2A*, *SLC1A4*, *ATF3,* and other core genes related to the mitral valve prolapse were screened out. *CDKN2A*, *SLC1A4,* and *ATF3* genes were at the core position of the network, regulated by numerous lncRNAs. Notably, these genes are primarily involved in the extracellular region and p53 signaling pathway.

**Conclusion:** In summary, *CDKN2A*, *SLC1A4*, and *ATF3* regulate the pathophysiological process of MVP and are potential therapeutic targets.

## Highlights

1 This is the first paper to investigate lncRNA in peripheral blood of MVP.
2 This study is the first to apply ferroptosis to the pathogenesis and treatment of MVP.
3 We are the first to identify genes and lncRNAs associated with ferroptosis in MVP, which is expected to be used in the diagnosis and treatment of this disease.

## Introduction

In European and American countries, degenerative mitral valve disease has an estimated incidence of 2.5%, replacing rheumatic valvular heart disease as an important public health threat (Pellerin et al., 2002). Degenerative mitral valve disease often manifests as lengthening or rupture of mitral chordae tendineae, causing mitral valve prolapse. The most common and currently available treatments for MVP include valve replacement and valve repair. Both treatments have their pros and cons. For instance, repaired valves do not require long-term anticoagulation, and there is a risk of reoperation. Suri et al, (2016) showed that a 15-year incidence of recurrent mitral regurgitation (grade 2 or greater) was 13.3% and that of reoperation was 6.9%. However, ideal preoperative or postoperative treatment to prevent MVP progression remains unavailable. Moreover, studies on the mechanism of MVP are limited. Therefore, there is a need to investigate the mechanism of MVP progression.

Ferroptosis is a modulated form of apoptosis, characterized by iron-dependent deposition of lipids and peroxides, causing programmed cell death due to intracellular lipid metabolism imbalance, iron homeostasis, and glutathione reduction (Hirschhorn and Stockwell, 2019). It is also a complex biological process mediated by several factors, including intracellular iron metabolism, glycolipid metabolism, and REDOX reactions (Li et al., 2020). In the human body, ferroptosis is involved in the occurrence and development of degenerative diseases, including Alzheimer’s disease, Parkinson’s disease, tumors, stroke, ischemia-reperfusion injury, etc (Li et al., 2019).

LncRNAs are non-coding RNAs with longer than 200 nucleotides (Bridges et al., 2021). Studies indicate that lncRNAs regulate gene expression dose compensation effect, epigenetic regulation, cell cycle regulation, and cell differentiation regulation (Batista and Chang, 2013; Tripathi et al., 2013).

Limited studies have so far reported the role of ferroptosis-related genes and lncRNAs in MVP. Herein, we performed transcriptome sequencing and obtained the results for differential gene analysis. This work is aimed at exploring the possible molecular mechanism of MVP and therapeutic target for the disease.

## Materials and methods

### Screening of patients with mitral valve prolapse and healthy volunteers

Study group: The inclusion criteria included: (1) Cardiac echocardiography revealed mitral valve prolapse with or without chordae rupture in patients; (2) Less than 40 mm left atrial diameter and less than 60 mm diastolic left ventricle diameter.

The exclusion criteria included: (1) Coronary CTA indicates single or multiple coronary artery stenosis; (2) Patients with other severe valvular diseases.

Healthy group: The inclusion criteria included: (1) Healthy individuals with normal cardiac structure diagnosed by cardiac echocardiography; (2) Undergo a physical examination and require a blood test. The exclusion criteria included: (1) Individuals under medications related to the cardiovascular system; (2) Individuals with a taste for tobacco and alcohol.

A total of five patients with mitral valve prolapse were recruited to the study group, whereas five volunteers were selected for the healthy group.

All patients and volunteers provided informed consent.

### RNA extraction and sequencing

Blood of patients with MVP before treatment and that of the healthy volunteers were collected to extract peripheral blood mononuclear cells (PBMCs) for RNA extraction.

RQ1 DNase (Promega) was used to remove the DNA of total RNA. The quality and quantity of the purified RNA were determined by measuring absorbance at 260nm/280 nm (A260/A280) using Smartspec plus (BioRad). RNA integrity was further confirmed by 1.5% agarose gel electrophoresis.

For each sample, 1 μg of total RNA was used for RNA-seq library preparation. mRNAs were captured by VAHTS mRNA capture Beads (Vazyme, N401). The purified RNA was treated with RQ1 DNase (Promega) to remove the DNA before directional RNA-seq. library preparation by KAPA Stranded mRNA-Seq Kit for Illumina® Platforms (KK8544). Polyadenylated mRNAs were purified and fragmented then the fragmented mRNAs were converted into double-stranded cDNA. After end repair and A tailing, DNAs were ligated to Diluted Roche Adaptor (KK8726). After purifying the ligation product and size fractioning to 300-500bps, the ligated products were amplified, purified, quantified, and stored at −80°C before sequencing. The strand marked with dUTP (the 2nd cDNA strand) was not amplified, allowing strand-specific sequencing.

For high-throughput sequencing, libraries were prepared as per the manufacturer’s instructions, then Illumina Novaseq 6000 system was used for 150 nt paired-end sequencing.

### mRNA expression statistics, lncRNA prediction, and lncRNA expression statistics

RNA-seq data were grouped, and StringTie was used to assemble each data group and predict the transcripts. The expression levels of the predicted transcripts were screened; transcripts with FPKM<1 were removed, before combining into one transcript using cuffcompare. Through CPC, CNCI, CPAT, and LGC analyses, the transcript was screened for coding potential and to establish whether the transcript is lncRNA. Notably, FPKM denotes the expected fragments per kilobase of transcript per million fragments mapped. The number of FPKM>0 and FPKM≥1 in lncRNA detected in this project was counted.

### LncRNA and mRNA differential analysis

Differentially expressed lncRNAs (DElncRNAs) and DEGs were obtained by comparing the predicted transcripts between samples. Raw reads were modeled using the DESeq2 software, and scale factors were used to explain the differences in the library depth. The DESeq2 software modeled the reads count by estimating gene dispersion and narrowing the estimates to generate accurate dispersion estimates. Eventually, the DESeq2 software fitted the model of the negative binomial distribution and used the Wald test or likelihood ratio test for hypothesis testing. Of note, the DESeq2 software analyzes the differential expression between two or more samples and establishes whether a gene is differentially expressed by fold change (FC) and correction P (FC ≥ 3/2 or ≤2/3, *p*-value < 0.01).

### Extraction of ferroptosis-related genes and construction of co-expression network

According to literature related to ferroptosis, 396 genes related to ferroptosis were obtained from the ferroptosis database (http://www.zhounan.org/ferrdb/legacy/index.html). The “Limma” package in R software was used to screen the ferroptosis-related genes and their expression levels in differential genes before drawing the heat maps. The Cytoscape software was used to construct the co-expression network of mRNAs and lncRNAs.

### Gene ontology analysis and kyoto encyclopedia of genes and genomes analysis

Gene Ontology (GO, http://geneontology.org/) is a database that describes the roles of genes and proteins. GO is divided into three categories, i.e., Molecular Function, Biological Process, and Cellular Component. On the other hand, the Kyoto Encyclopedia of Genes and Genomes (KEGG, http://www.kegg.jp/) is a database resource for understanding advanced functional and biological systems from molecular-level information, particularly genome sequencing and other high-throughput experimental techniques generated from large molecular datasets. The roles of LncRNA targets were analyzed *via* GO classification and enrichment analyses. First, lncRNA targets were mapped to each term in the GO database, before counting the number of genes in each term. Subsequently, a hypergeometric distribution test was used to obtain the GO term with significant enrichment of lncRNA targets against the background of GO annotation of the whole genome. The KEGG pathway enrichment analysis was performed on the lncRNA targets to analyze their functions. First, lncRNA targets were mapped to each pathway in the KEGG database before counting the number of genes in each pathway. Subsequently, the KEGG pathway with significantly enriched lncRNA targets was obtained using a hypergeometric distribution test against the background of KEGG annotation of the whole genome. We then obtained the intersection of screened ferroptosis-related genes with GO enrichment analysis and KEGG pathway enrichment analysis results.

### Data analysis

SPSS25.0 software was used for data analysis, and R software (version 4.1.2) was used for image rendering.

## Results

### DEGs and DElncRNAs

**FIGURE 1:**
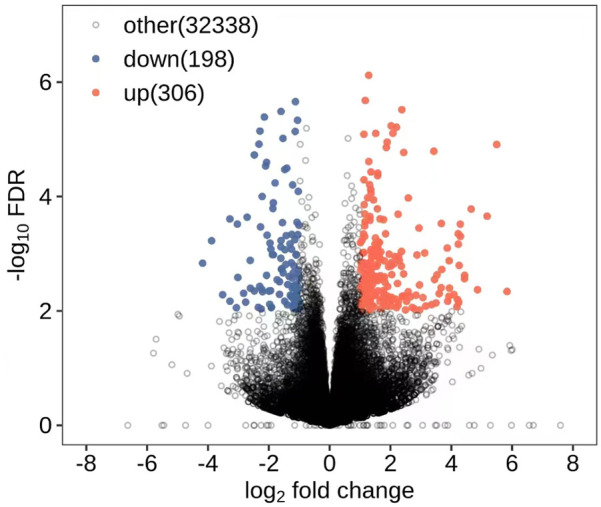
DEGs.

**FIGURE 2:**
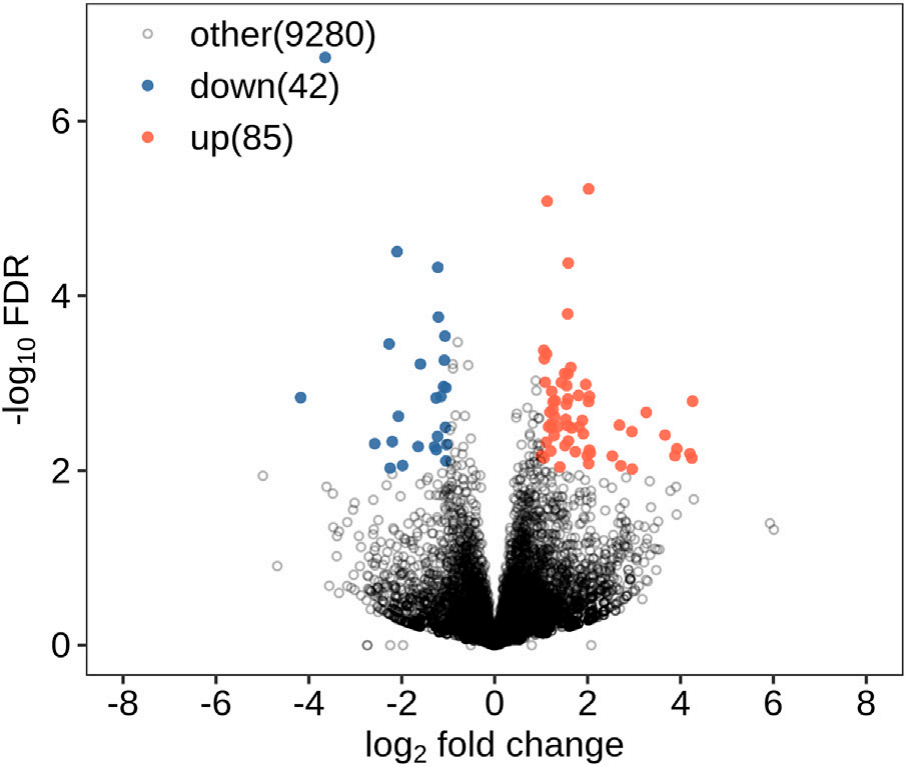
DElncRNAs.

All DEGs and DElncRNAs were screened by comprehensive bioassay. A total of 504 DEGs (Figure 1) and 127 DElncRNAs (Figure 2) were screened. Among these, 198 DEGs were upregulated, 306 were downregulated; 85 DElncRNAs were upregulated, and 42 DElncRNAs were downregulated.

### Extraction of differentially expressed ferroptosis-related genes and construction of co-expression network

**FIGURE 3:**
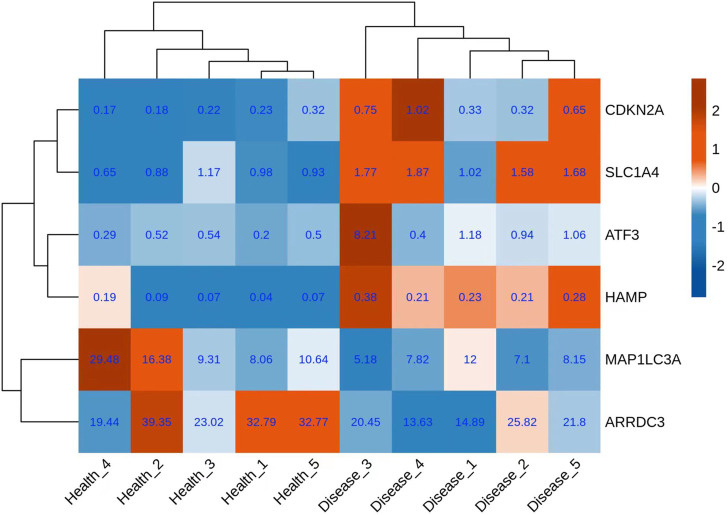
Expression of differential genes associated with ferroptosis.

**FIGURE 4:**
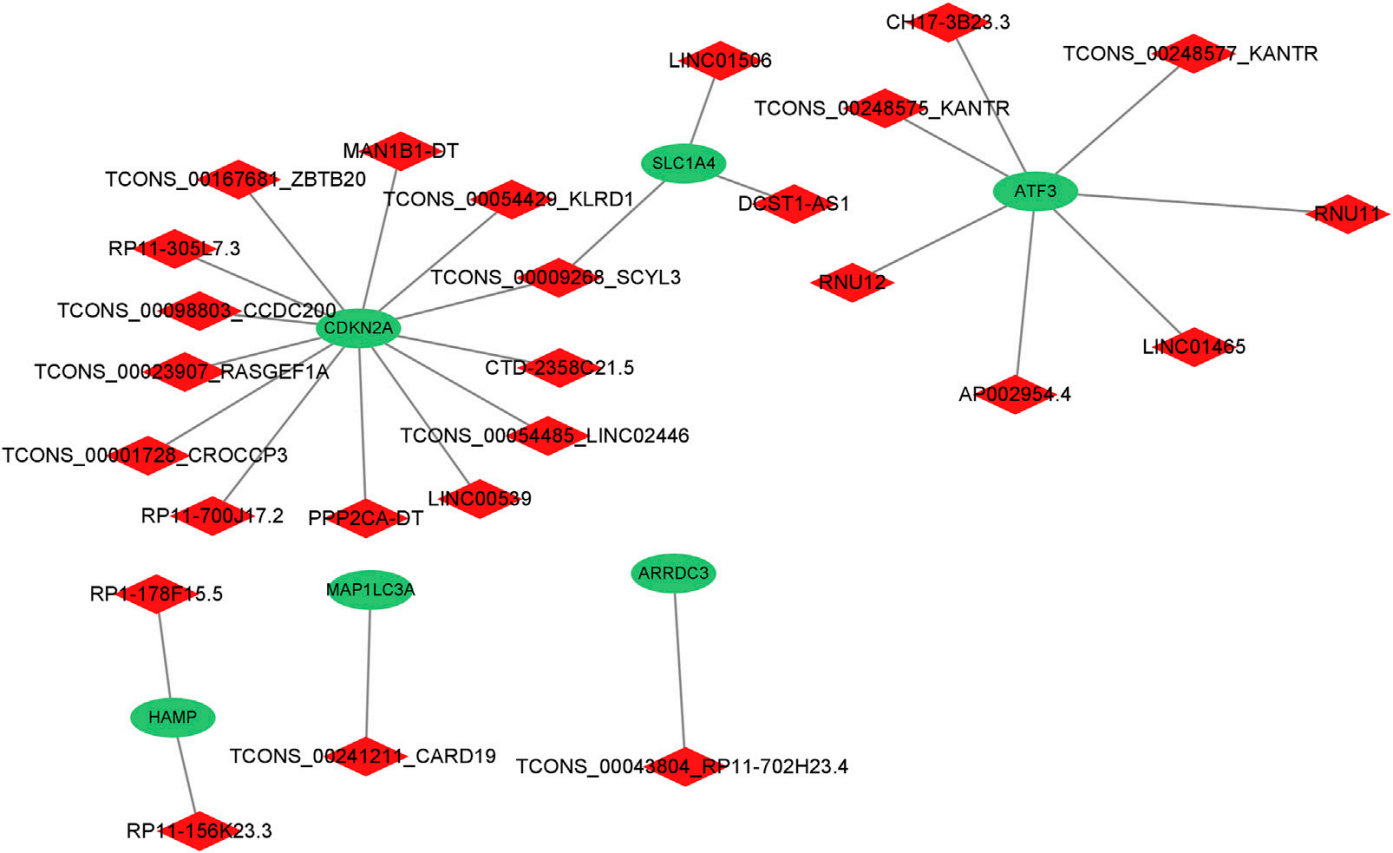
The co-expression relationship network diagram about differential genes related to ferroptosis and the differential lncRNAs co-expressed with the differential genes related to ferroptosis.

A total of six genes related to ferroptosis were extracted (Figure 3); co-expression analysis results showed 27 co-expressed differential lncRNAs with differential ferroptosis-related genes. The co-expression relationship network diagram was constructed after inputting the above-mentioned differential ferroptosis-related genes and differential lncRNAs co-expressed with differential ferroptosis-related genes into the Cytoscape software (Figure 4). Consequently, *CDKN2A*, *SLC1A4,* and *ATF3* genes were in the network core location and co-expressed with multiple lncRNAs like LINC01465, LINC00539, and TCONS_00009268_SCYL.

### Functional enrichment of Gene Ontology in DEGs

**FIGURE 5:**
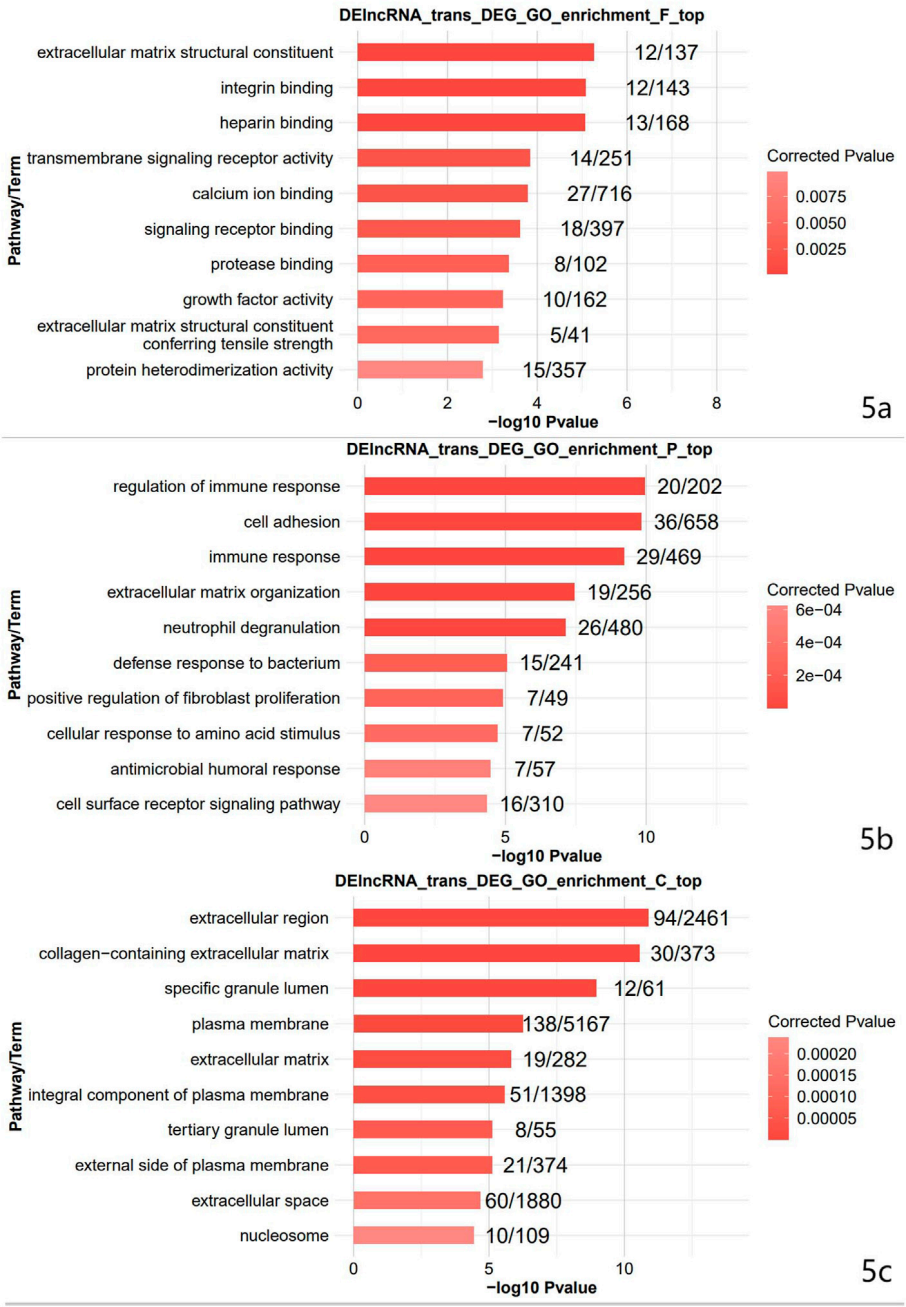
The result of GO enrichment analysis. GO is divided into Molecular Function (Figure 5A), Biological Process (Figure 5B), and Cellular Component (Figure 5C).

The top 10 terms were selected for display. The top 10 terms of molecular function included: extracellular matrix structural constituent, integrin binding, heparin-binding, transmembrane signaling receptor activity, calcium ion binding, signaling receptor binding, protease binding, growth factor activity, extracellular matrix structural constituent conferring tensile strength, and protein heterodimerization activity (Figure 5A). The top 10 terms of the biological process included: immune response regulation, cell adhesion, immune response, extracellular matrix organization, neutrophil degranulation, defense response to a bacterium, positive regulation of fibroblast proliferation, cellular response to amino acid stimulus, antimicrobial humoral response, and cell surface receptor signaling pathway (Figure 5B). The top 10 terms of cellular component included: extracellular region, collagen-containing extracellular matrix, specific granule lumen, plasma membrane, extracellular matrix, an integral component of the plasma membrane, tertiary granule lumen, external side of the plasma membrane, extracellular space, and nucleosome (Figure 5C). Differential ferroptosis-related genes were primarily enriched in the extracellular region, plasma membrane, an integral component of the plasma membrane and extracellular cell space, protein heterodimerization activity, immune response, and defense response to a bacterium.

### Signal pathway enrichment in DEGs

**FIGURE 6:**
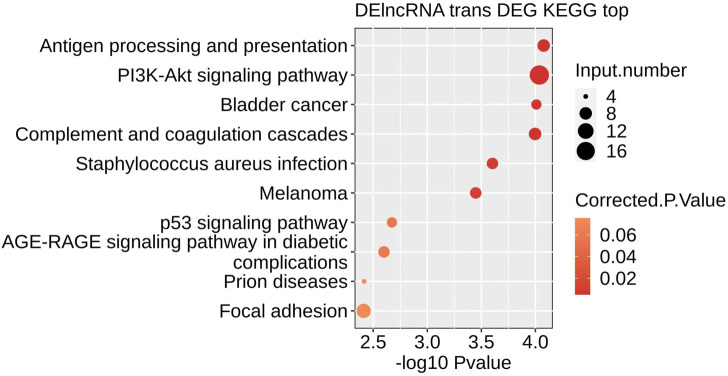
The result of KEGG enrichment analysis.

The top 10 Pathways included: Antigen processing and presentation, PI3K-Akt signaling pathway, Bladder cancer, Complement, and coagulation cascades, *Staphylococcus aureus* infection, Melanoma, p53 signaling pathway, AGE−RAGE signaling pathway in diabetic complications, Prion diseases, Focal adhesion (Figure 6). KEGG signaling pathway analysis revealed that different ferroptosis-related genes are primarily involved in the p53 signaling pathway.

## Discussion

This work discovered *CDKN2A*, *SLC1A4,* and *ATF3* genes in the network core position, regulated by numerous lncRNAs correlating with each other. This indicates that these genes regulate the pathophysiological process of rupture in mitral chordae tendineae.

So far, the relationship between lncRNAs and MVP remains unknown. We found 127 DElncRNAs, indicating that lncRNAs are involved in pathogenesis of MVP. Previous studies found lncRNAs to be differentially expressed in valvular interstitial cells (VICs) exposed to a higher strain (Wu et al., 2019; Gupta et al., 2020). In MVP, mitral regurgitation causes the mitral chordae tendineae to remain in a constant state of high tension; this explains the differential expression of lncRNAs.

*CDKN2A* gene is involved in the regulation of cell proliferation, cell collapse, and apoptosis (Kim and Sharpless, 2006). Previous studies have reported bidirectional interaction of *CDKN2A* gene expression. Knösel et al, (2014) reported that *CDKN2A* upregulation is linked to oncogene-induced aging, whereas *CDKN2A* loss potentially triggers malignant progression. Additionally, Bayoglu et al, (2014) reported that upregulated *CDKN2A* gene expression in arterial plaques increases the risk of atherosclerosis and promotes carotid stenosis development.

*The SLC1A4* gene is a sodium-dependent neutral amino acid transporter involved in various pathophysiological processes including tumorigenesis (Peng et al., 2021). According to Wu et al, (2022), *SLC1A4* upregulation modulates the occurrence and progression of coronary atherosclerosis. The *SLC1A4* gene is a potential biomarker and therapeutic target for coronary atherosclerosis.

Activated transcription factor 3 (*ATF3*) belongs to the *ATF*/*CREB* transcription factor family; its expression is rapidly induced by various cellular stresses, including DNA damage, oxidative stress, and cell damage (Wang et al., 2020). Kalfon et al, (2019) found that mice with *ATF3* gene defects experience maladaptive heart remodeling and reduced cardiac hypertrophy. Therefore, treatment targeting *ATF3* activity may be beneficial for the recovery of cardiac function.

*HAMP* regulates systematic iron metabolism and anemia of inflammation. It induces hemochromatosis, secondary anemia, and non-HFE forms of hemochromatosis, which is related to the TGF-beta signaling pathway (Wang and Du, 2021). According to recent reports, upregulated *HAMP* expression indicates poor cancer prognosis, including non-small cell lung and prostate cancers (Chen et al., 2014; Tesfay et al., 2015). However, a few studies have reported a relationship between this gene and cardiac disease. This study discovered *HAMP* upregulation in patients with MVP, indicating that this gene can predict the prognosis of MVP.

*ARRDC3* and *MAP1LC3A* genes were downregulated in this work. Zhang et al, (2022) found that *ARRDC3* potentially inhibits liver fibrosis and epithelial-to-mesenchymal transition, which is related to the *ITGB4/PI3K/Akt* signaling pathway. GO analysis results showed that extracellular matrix deposition, specifically collagen fiber is a key pathogenic mechanism of MVP. Thus, we believe that the *ARRDC3* gene inhibits fibrosis in diseased chordae tendineae, consequently suppressing collagen fiber deposition. *MAP1LC3A* encodes microtubule-associated protein one light chain three alpha (LC3), which reflects autophagic activity (Qin et al., 2021). Therefore, autophagy may be an important molecular event in the pathogenesis of MAP1L3A. Besides, the downregulation of *ARRDC3* and *MAP1LC3A* genes confirms their protective effects in MVP.

According to the GO and KEGG analyses, the core genes mentioned above are primarily involved in the extracellular region and p53 signaling pathway; this may explain the initial factors of MVP. MVP is characterized by myxoid degeneration of leaflets and chordae tendineae, characterized by leaflet thickening and extracellular matrix deposition (Guy and Hill, 2012). Kim et al, (2020) revealed that although heart valves primarily comprise valvular endothelial and valve interstitial cells, white blood cells, including CD45^+^ cells, can also be detected between thickened valve tissue in normal and diseased valves in mice and humans. Therefore, a sterile proinflammatory microenvironment promotes the development of MVP.

Abnormal iron metabolism in inflammation causes intracellular iron overload, thereby promoting the polarization of M1 macrophages (Zhou et al., 2018). Iron regulates ferroptosis and the immune system. Ferroptosis is a recently discovered form of iron-dependent cell death. This form of cell death does not exhibit typical morphological and biochemical characteristics, including cell contraction, mitochondrial fragmentation, and nuclear concentration. The three basic features of ferroptosis include lipid peroxides clearance dysfunction, the presence of REDOX active iron, and peroxidation of phospholipids with polyunsaturated fatty acids (Tang and Kroemer, 2020; Tang et al., 2021). (Jiang et al., 2021; Tang et al., 2021).

In the inflammatory microenvironment of mitral valve prolapse, abnormal iron metabolism causes programmed cell death of mitral valve interstitial cells, abnormal secretion of extracellular matrix, and reconstruction of extracellular matrix, which may be mechanisms of mitral valve prolapse. Furthermore, accumulating evidence suggests that ferroptosis is related to several cardiovascular diseases, including atherosclerosis, stroke, ischemia-reperfusion injury, and heart failure. This indicates a signal for ferroptosis-related gene targeting therapy; thus, inhibiting cardiac cell death provides novel therapy for cardiovascular disease (Fang et al., 2020; Li et al., 2021; Yu et al., 2021).

## Conclusion

In conclusion, *CDKN2A*, *SLC1A4*, and *ATF3* genes modulate the pathogenesis of MVP. We believe that targeted silencing of these genes can slow down the pathogenesis of MVP, thereby delaying disease occurrence and development as well as improving the prognosis of patients.

## Limitation

This study used a small blood sample size, therefore, more specimens are necessary to validate the current results and identify more DEGs. Moreover, an ideal animal model of MVP is unavailable across the globe, thus presenting a challenge in conducting animal experiments.

## Data Availability

The raw data supporting the conclusion of this article will be made available by the authors, without undue reservation.
